# Supplementary material for: Comparison of low-salt preference trends and regional variations between patients with major non-communicable diseases and the general population
Source: PLoS One. 2022 Oct 25;17(10):e0276655. doi: 10.1371/journal.pone.0276655 (PMC9595509; doi:10.1371/journal.pone.0276655)
Supplement: S1 File — (PDF) [file pone.0276655.s001.pdf]

Supplementary File 1. Regional variations in low-salt preference (as of 2019, dyslipidaemia as of 2017)

1. Overall low-salt preference

Region	Hypertension	Diabetes	Dyslipidaemia	General population
Total	23.6%	24.6%	24.2%	21.6%
Seoul	27.3%	27.3%	24.2%	24.3%
Busan	24.5%	25.9%	27.2%	22.0%
Daegu	24.9%	26.9%	26.7%	23.7%
Incheon	23.0%	24.5%	22.2%	19.9%
Gwangju	22.6%	22.9%	21.5%	18.4%
Daejun	23.0%	24.8%	26.3%	21.0%
Ulsan	23.3%	26.4%	23.2%	22.8%
Sejong	23.8%	28.0%	26.1%	20.5%
Gyeonggi	22.1%	23.3%	23.1%	20.8%
Gangwon	21.6%	23.5%	23.2%	20.4%
Chungbuk	21.8%	27.2%	23.9%	21.5%
Chungnam	22.9%	23.2%	25.5%	21.2%
Jeonbuk	21.0%	21.8%	25.0%	18.4%
Jeonnam	21.8%	22.7%	21.7%	18.3%
Gyeongbuk	24.1%	25.3%	25.7%	22.4%
Gyeongnam	21.5%	20.8%	27.5%	21.8%
Jeju	29.1%	30.1%	20.5%	23.0%
Standard deviation	2.1%	2.4%	2.1%	1.7%

2. Low-salt preference at the table

Region	Hypertension	Diabetes	Dyslipidaemia	General population
Total	68.1%	68.3%	67.2%	68.4%
Seoul	72.2%	72.9%	69.0%	70.8%
Busan	67.3%	67.3%	69.1%	66.5%
Daegu	59.2%	60.7%	57.8%	61.8%
Incheon	71.7%	69.4%	71.4%	71.9%
Gwangju	81.1%	82.1%	78.6%	77.0%
Daejun	58.2%	57.5%	68.6%	54.1%
Ulsan	69.2%	68.9%	70.7%	72.8%
Sejong	64.5%	64.5%	58.9%	70.4%
Gyeonggi	68.1%	67.5%	66.4%	68.5%
Gangwon	63.7%	65.3%	65.6%	64.1%
Chungbuk	67.3%	66.4%	68.9%	70.1%
Chungnam	60.2%	63.3%	57.1%	60.5%
Jeonbuk	62.8%	64.5%	66.7%	64.2%
Jeonnam	76.2%	74.6%	66.3%	75.9%
Gyeongbuk	64.4%	67.0%	60.7%	67.7%
Gyeongnam	69.8%	68.7%	72.4%	71.5%
Jeju	67.2%	60.1%	70.6%	62.9%
Standard deviation	5.8%	5.6%	5.5%	5.7%

3. Low-salt preference for fried food

Region	Hypertension	Diabetes	Dyslipidaemia	General population
Total	44.9%	45.2%	40.5%	36.4%
Seoul	47.3%	49.5%	39.7%	37.9%
Busan	47.8%	47.6%	46.1%	36.5%
Daegu	31.2%	29.4%	26.8%	23.6%
Incheon	45.2%	48.2%	38.8%	35.8%
Gwangju	60.9%	59.6%	49.0%	40.7%
Daejun	43.0%	45.6%	42.1%	35.0%
Ulsan	35.5%	39.2%	35.1%	29.5%
Sejong	28.6%	20.3%	31.6%	31.2%
Gyeonggi	42.0%	42.5%	39.1%	35.2%
Gangwon	30.9%	30.4%	35.8%	27.8%
Chungbuk	38.3%	39.2%	37.2%	32.5%
Chungnam	47.0%	46.7%	42.7%	37.8%
Jeonbuk	57.1%	52.1%	52.7%	47.4%
Jeonnam	67.0%	65.0%	54.0%	55.8%
Gyeongbuk	30.0%	28.1%	31.6%	25.0%
Gyeongnam	53.7%	53.3%	52.4%	44.4%
Jeju	50.8%	49.6%	37.4%	43.2%
Standard deviation	10.9%	11.4%	7.7%	8.0%

4. Law-salt preference rate (type III)

Region	Hypertension	Diabetes	Dyslipidaemia	General population
Total	12.8%	13.7%	12.4%	10.4%
Seoul	15.6%	16.8%	12.6%	12.3%
Busan	13.6%	14.0%	14.6%	10.3%
Daegu	9.6%	10.4%	6.7%	7.6%
Incheon	13.3%	15.1%	10.5%	10.0%
Gwangju	15.3%	14.6%	13.4%	10.3%
Daejun	11.7%	14.9%	13.7%	10.0%
Ulsan	10.4%	12.7%	10.5%	9.9%
Sejong	10.4%	8.9%	13.0%	7.7%
Gyeonggi	12.1%	13.4%	11.6%	10.0%
Gangwon	9.7%	8.6%	11.2%	8.5%
Chungbuk	10.9%	13.7%	13.2%	10.1%
Chungnam	11.9%	12.3%	11.0%	10.2%
Jeonbuk	13.2%	13.4%	15.4%	10.4%
Jeonnam	15.8%	17.0%	13.9%	12.1%
Gyeongbuk	9.6%	10.2%	11.7%	8.1%
Gyeongnam	12.3%	11.7%	18.1%	11.8%
Jeju	17.0%	16.2%	11.2%	11.0%
Standard deviation	2.3%	2.5%	2.4%	1.4%
