# Supplementary material for: Comparison of low-salt preference trends and regional variations between patients with major non-communicable diseases and the general population
Source: PLoS One. 2022 Oct 25;17(10):e0276655. doi: 10.1371/journal.pone.0276655 (PMC9595509; doi:10.1371/journal.pone.0276655)
Supplement: S2 File — (PDF) [file pone.0276655.s002.pdf]

Regional characteristics related to sodium intake

Region	Si or Do	Sodium intake*	fat intake*	Average temperature**	Precipitation***
Seoul	Si	3821.9	51.3	13.2	891.3
Busan	Si	3716.3	54.6	15.2	1623.2
Daegu	Si	3910.0	49.5	14.5	995.7
Incheon	Si	3767.2	49.7	12.3	864.7
Gwangju	Si	4073.9	50.6	14.5	1085.9
Daejeon	Si	3422.1	39.2	13.7	984.2
Ulsan	Si	3655.1	45.1	14.6	1450.1
Sejong	Si	3441.0	48.4	13.1	959.3
Gyeonggi	Do	3752.5	49.8	12.2	1000.5
Gangwon	Do	3787.3	50.8	11.7	1094.6
Chungbuk	Do	4383.7	49.8	12.1	941.1
Chungnam	Do	3990.1	55	12.7	913.2
Jeonbuk	Do	3825.4	50.4	13.0	1148.2
Jeonnam	Do	3514.2	53.7	14.1	1401.9
Gyeongbuk	Do	3217.7	35.2	13.0	1147.8
Gyeongnam	Do	4134.3	56.3	14.1	1588.4
Jeju	Do	4401.2	58.3	16.4	2102.3

* https://www.khidi.or.kr/nns/CityNutriStat/info?menuId=MENU02559

** https://kosis.kr/statHtml/statHtml.do?orgId=101&tblId=DT_1YL9801

*** https://kosis.kr/statHtml/statHtml.do?orgId=101&tblId=DT_1YL9901
