# Supplementary material for: Comparison of low-salt preference trends and regional variations between patients with major non-communicable diseases and the general population
Source: PLoS One. 2022 Oct 25;17(10):e0276655. doi: 10.1371/journal.pone.0276655 (PMC9595509; doi:10.1371/journal.pone.0276655)
Supplement: Attachment — Submitted filename: Review comments response_20220923.docx [file pone.0276655.s003.docx]

PONE-D-22-18808

Comparison of low-salt preference trends and regional variations between patients with major non-communicable diseases and the general population

PLOS ONE

Dear Dr. Ock,

Thank you for submitting your manuscript to PLOS ONE. After careful consideration, we feel that it has merit but does not fully meet PLOS ONE’s publication criteria as it currently stands. Therefore, we invite you to submit a revised version of the manuscript that addresses the points raised during the review process.

==============================

Two reviewers well assessed this manuscript.

However, there are still corrections existed in the present form of the manuscript.

Read the suggestions carefully and respond them appropriately.

==============================

Please submit your revised manuscript by Sep 28 2022 11:59PM. If you will need more time than this to complete your revisions, please reply to this message or contact the journal office at plosone@plos.org. When you're ready to submit your revision, log on to https://www.editorialmanager.com/pone/ and select the 'Submissions Needing Revision' folder to locate your manuscript file.

Please include the following items when submitting your revised manuscript:

A rebuttal letter that responds to each point raised by the academic editor and reviewer(s). You should upload this letter as a separate file labeled 'Response to Reviewers'.

A marked-up copy of your manuscript that highlights changes made to the original version. You should upload this as a separate file labeled 'Revised Manuscript with Track Changes'.

An unmarked version of your revised paper without tracked changes. You should upload this as a separate file labeled 'Manuscript'.

If you would like to make changes to your financial disclosure, please include your updated statement in your cover letter. Guidelines for resubmitting your figure files are available below the reviewer comments at the end of this letter.

If applicable, we recommend that you deposit your laboratory protocols in protocols.io to enhance the reproducibility of your results. Protocols.io assigns your protocol its own identifier (DOI) so that it can be cited independently in the future. For instructions see: https://journals.plos.org/plosone/s/submission-guidelines#loc-laboratory-protocols. Additionally, PLOS ONE offers an option for publishing peer-reviewed Lab Protocol articles, which describe protocols hosted on protocols.io. Read more information on sharing protocols at https://plos.org/protocols?utm_medium=editorial-email&utm_source=authorletters&utm_campaign=protocols.

We look forward to receiving your revised manuscript.

Kind regards,

Masaki Mogi

Academic Editor

PLOS ONE

Journal Requirements:

When submitting your revision, we need you to address these additional requirements.

1. Please ensure that your manuscript meets PLOS ONE's style requirements, including those for file naming. The PLOS ONE style templates can be found at

https://journals.plos.org/plosone/s/file?id=wjVg/PLOSOne_formatting_sample_main_body.pdf and

https://journals.plos.org/plosone/s/file?id=ba62/PLOSOne_formatting_sample_title_authors_affiliations.pdf

**Response: We rechecked whether the submitted manuscript followed the guidelines.**

2. Thank you for stating in your Funding Statement:

"This work was supported by the Research Program funded by the Korea Disease Control and Prevention Agency. The funders had no role in study design, data collection and analysis, decision to publish, or preparation of the manuscript."

Please provide an amended statement that declares *all* the funding or sources of support (whether external or internal to your organization) received during this study, as detailed online in our guide for authors at http://journals.plos.org/plosone/s/submit-now. Please also include the statement “There was no additional external funding received for this study.” in your updated Funding Statement.

Please include your amended Funding Statement within your cover letter. We will change the online submission form on your behalf.

**Response: As pointed out, we have modified the Funding Statement.**

3. Please review your reference list to ensure that it is complete and correct. If you have cited papers that have been retracted, please include the rationale for doing so in the manuscript text, or remove these references and replace them with relevant current references. Any changes to the reference list should be mentioned in the rebuttal letter that accompanies your revised manuscript. If you need to cite a retracted article, indicate the article’s retracted status in the References list and also include a citation and full reference for the retraction notice.

**Response: We double-checked that we cited references correctly.**

Reviewers' comments:

Reviewer's Responses to Questions

Comments to the Author

1. Is the manuscript technically sound, and do the data support the conclusions?

The manuscript must describe a technically sound piece of scientific research with data that supports the conclusions. Experiments must have been conducted rigorously, with appropriate controls, replication, and sample sizes. The conclusions must be drawn appropriately based on the data presented.

Reviewer #1: Yes

Reviewer #2: Yes

2. Has the statistical analysis been performed appropriately and rigorously?

Reviewer #1: Yes

Reviewer #2: I Don't Know

3. Have the authors made all data underlying the findings in their manuscript fully available?

The PLOS Data policy requires authors to make all data underlying the findings described in their manuscript fully available without restriction, with rare exception (please refer to the Data Availability Statement in the manuscript PDF file). The data should be provided as part of the manuscript or its supporting information, or deposited to a public repository. For example, in addition to summary statistics, the data points behind means, medians and variance measures should be available. If there are restrictions on publicly sharing data—e.g. participant privacy or use of data from a third party—those must be specified.

Reviewer #1: Yes

Reviewer #2: Yes

4. Is the manuscript presented in an intelligible fashion and written in standard English?

PLOS ONE does not copyedit accepted manuscripts, so the language in submitted articles must be clear, correct, and unambiguous. Any typographical or grammatical errors should be corrected at revision, so please note any specific errors here.

Reviewer #1: Yes

Reviewer #2: Yes

5. Review Comments to the Author

Please use the space provided to explain your answers to the questions above. You may also include additional comments for the author, including concerns about dual publication, research ethics, or publication ethics. (Please upload your review as an attachment if it exceeds 20,000 characters)

Reviewer #1: Dear Authors,

The article provides a good insight into the trends in low-salt preference and regional differences in Korea among patients with the main non-communicable diseases.

For a better understanding of the work, I have some suggestions:

**Response: We really appreciate the careful and thorough review of this manuscript and for the thoughtful comments and constructive suggestions, which help to improve the quality of the manuscript. Our responses are as follows.**

Line 118.

Can the authors explain the background or give a reference why the 13 NCD listed are the main ones?

**Response: Thirteen major types of NCD patients (hypertension, diabetes, dyslipidaemia, stroke, myocardial infarction, angina, arthritis, osteoporosis, asthma, allergic rhinitis, atopic dermatitis, cataract, and depression) were defined as participants who responded clinically diagnosed from physicians in the KCHS. We have revised the sentences (line 117-120).**

Line 208:

To the reader who does not know Korea well, the names of the regions do not give useful information.

Would it make sense to describe each region (in few words) background that could influence salt consumption?

For example,

-a coastal region, traditionally lot of fish are consumed,…

-an inland region, …

-rural region,... intensive urban region,..

-a region where traditionally few meat is consumed...

**Response: Thanks for the constructive suggestion. As suggested, we have added regional characteristics of Republic of Korea, including sodium intake, as a Supplemental file. Sentences that further research on regional variation are needed have been added to the Discussion section (Line 292-297). However, we ask for your understanding that we cannot cover various regional characteristics due to data limitations.**

Lines 234 235:

The low-salt preference rates of patients with different major NCDs and the general population was gradually decreasing after peaking mainly in 2013

The trend is evident from figures 2,3,4,5, can you comment the reasons for the trend?

**Response: In Republic of Korea, the multicomponent national programs to reduce salt intake has been steadily promoted since the 2010s. In particular, in 2012, a national plan was established to reduce sodium intake. However, we estimated that this effect appears to have gradually weakened. These contents were further reinforced in the Discussion section (Line 256-259).**

Line 305:

In the title of the article you "regional variations" are "highlighted

Can you briefly comment the reasons for the differences in patient behavior between regions?

**Response: As mentioned in the previous response, the need for additional studies on regional variation was emphasized in the Discussion section (Line 292-297).**

Reviewer #2: This study deals with an important issue within Korea as well as worldwide. The study uses the results of a large survey to analyze trends over time in the differences in low salt preference between major NCD patients and the general population, and offers suggestions for future environmental improvement and education. However, there are some concerns in this paper.

**Response: We really appreciate the careful and thorough review of this manuscript and for the thoughtful comments and constructive suggestions, which help to improve the quality of the manuscript. Our responses are as follows.**

1. Abstract: Result: The authors specifically describe the low salt preference rate. Since the survey years for this study are 2008-2019, Please indicate the year of the survey.

**Response: In the Materials and methods section, we have already mentioned that the Korea Community Health Survey is conducted annually (Line 110). The Korea Community Health Survey results for each year were used for analysis.**

2. Introduction: 87: The survey years are 2008-2017, but is it 2008-2019?

**Response: In the previous study (Reference number 10), the results of the Korea Community Health Survey from 2008 to 2017 were analyzed to analyze health behaviors, such as smoking and drinking, among major NCD patients. This content is a description of prior study.**

3. Materials and methods: What is the size of The Korea Community Health Survey? Please provide sample size information for each survey year.

**Response: As suggested, we added information about sample size from the Korea Community Health Survey to the Materials and methods section (Line 113-114).**

4.Materials and methods: Participants: The authors do not include cancer as Major NCD Patients, although they mention it in the study limitations. Why not include cancer in this analysis?

**Response: Unfortunately, the Korea Community Health Survey does not include a question asking whether participants have cancer.** **We emphasize this point further in the part on limitations in the Discussion section (Line 312-314).**

In addition, since the results and discussion focus on hypertension, diabetes, and dyslipidemia, it would be better to exclude other diseases. I think it would be better to have the data of Fig2-Fug5 to grasp the overall trend of NCD, but I feel it is difficult to confirm the data.

**Response: As you know, among various NCDs, hypertension, diabetes, and dyslipidemia are important diseases that act as risk factors for other diseases. Also, these diseases have a higher prevalence than other diseases. So, Figure 1 focused on hypertension, diabetes, and dyslipidemia. In addition, a Supplemental file 1 is attached so that readers can check the specific values for each disease.**

5.Results:178-179:　“In the general population, it peaked in 2013 at 74.4% (APC: -11.788, p-value: <0.001)”. Isn't the APC value 11.788?

**Response: As you pointed out, this is a typo. We have corrected that value (Line 182).**

6.Results:184-185：Other NCD results are not mentioned in the results that follow. If the relevant data set is not always available, then it may be possible to remove it.

**Response: As suggested, we have deleted that sentence.**

7. Results:194：Please list the ACP values for 2013.

**Response: It has a single APC with respect to its content. That is, the APC is -0.152.**

8. Results:201：Please correct the p-value.

**Response: We have modified the p-value (Line 202).**

9. Discussion: The low salt preference rate and the adherence rate of not adding salt or soy sauce at the table have changed since 2013, but the reasons for this have not been mentioned. Is there any possible reason for this change, such as a change in the survey methodology, the selection method of the target population, or a change in government policy policy regarding salt reduction?

**Response: In Republic of Korea, the multicomponent national programs to reduce salt intake has been steadily promoted since the 2010s. In particular, in 2012, a national plan was established to reduce sodium intake. However, we estimated that this effect appears to have gradually weakened. These contents were further reinforced in the Discussion section (Line 256-261).**

10. Discussion：260-261：“the reaction to a salty taste is influenced by the sodium concentration in the food”. It would be helpful to the reader's understanding if you could describe the specific impacts while presenting the reports of the cited references.

**Response: As suggested, we revised the sentence (line 265-266).**

11. Discussion：260-261：Please describe in detail the contents of your education to cooks and foodservice professionals.

**Response: The content of the sentence you pointed out has been modified to be more specific (Line 286-287).**

12. Disccussion：287-288：Please cite any reports showing that low-salt preference is related to temperature.

**Response: We have corrected the sentence and added a sentence that additional research on regional variation is needed (Line 292-297).**

6. PLOS authors have the option to publish the peer review history of their article (what does this mean?). If published, this will include your full peer review and any attached files.

If you choose “no”, your identity will remain anonymous but your review may still be made public.

Do you want your identity to be public for this peer review? For information about this choice, including consent withdrawal, please see our Privacy Policy.

Reviewer #1: No

Reviewer #2: No
